# The Relationship Among Internet Addiction, Depression, and Anxiety in Children and Adolescents: A Network Analysis

**DOI:** 10.1155/da/3015380

**Published:** 2026-11-01

**Authors:** Ya Wang, Han Wang, Jing Guo, Jia Huang, Jing Xiao, Qi Li

**Affiliations:** ^1^ School of Psychology Capital Normal University Beijing China cnu.edu.cn; ^2^ Neuropsychology and Applied Cognitive Neuroscience Laboratory State Key Laboratory of Cognitive Science and Mental Health Institute of Psychology Chinese Academy of Sciences Beijing China cas.cn; ^3^ Center for Mental Health Applications Institute of Psychology Chinese Academy of Sciences Beijing China cas.cn; ^4^ Department of Psychology The University of Chinese Academy of Sciences Beijing China ucas.ac.cn; ^5^ College of Education Capital Normal University Beijing China cnu.edu.cn; ^6^ Beijing Key Laboratory of Learning and Cognition and Department of Psychology Capital Normal University Beijing China cnu.edu.cn

**Keywords:** age differences, anxiety, depression, internet addiction, network analysis

## Abstract

**Background:**

With the increasing prevalence of internet use among younger age groups, internet addiction and its associated mental health problems, particularly depression and anxiety, have attracted increasing public attention. Although studies have revealed an association between internet addiction and mental health, there is still a lack of in‐depth understanding of the interactions among specific symptoms and differences between children and adolescents. A network analysis approach was adopted to address these issues.

**Methods:**

A total of 12,779 participants aged 10–18 years completed the Internet Addiction Test (IAT), Patient Health Questionnaire‐9 (PHQ‐9), and Generalized Anxiety Disorder‐7 (GAD‐7) through an online platform. Symptom networks of internet addiction, depression, and anxiety were constructed separately for children and adolescents and compared between the two groups.

**Results:**

The most central symptoms were consistent across the two groups (children and adolescents), including “*anticipation*” (IAT11), “*neglect chores to spend more time online*” (IAT2), and “*uncontrollable worrying*” (GAD2). Bridge symptoms were also similar across the two groups, including “*nervousness*” (GAD1), “*irritability*” (GAD6), and “*restlessness*” (GAD5). However, there were significant differences in network structure, with adolescents exhibiting stronger within‐community (within‐syndrome) edges and children exhibiting stronger between‐community (cross‐syndrome) edges.

**Conclusions:**

This study revealed similarities and differences in the network structures of internet addiction, depression, and anxiety between children and adolescents. These findings may provide a basis for targeted intervention strategies.

## 1. Introduction

Internet users are becoming younger and younger. According to the 53rd Statistical Report on the Development of the Internet in China, the number of underage internet users in China was 202 million, and internet users aged 10–19 account for 14.7% of the total [1]. The prevalence of internet addiction in this age group was 10.32% [2]. Internet addiction is defined as a psychological dependence on the internet, regardless of the type of activity after logging on. Specifically, it refers to the inability to control one’s internet use, which leads to negative effects in various areas of life, such as physical health problems [3], mental health problems [4], learning problems [5], and interpersonal problems [6].

Studies have shown that internet addiction coexists with mental health problems such as anxiety and depression [7]. Anxiety and depression are also two common mental health problems in the age period of 10–18 years [8], with the prevalence of anxiety and depression being 8.30% and 6.56%, respectively [9].

According to the cognitive behavioral model of internet addiction and compensatory internet use theory [10–12], negative emotions may trigger excessive internet use, which may lead to worse consequences, including increased depression and anxiety. Therefore, the relationship between addictive behaviors and mental health problems is bidirectional. The vicious cycle may result in the co‐occurrence of internet addiction and psychiatric symptoms (such as anxiety and depression) [13].

In traditional psychopathological studies, total scores of standardized measures are often used to indicate the severity of psychiatric symptoms. However, this approach may mask substantial differences between individual symptoms and the interaction between symptoms [14]. Network analysis is a new approach that has been used to examine the interrelationships between symptoms; this method has the advantage of examining the relationship between two nodes while controlling for all other nodes in the network [15]. In the network model, symptoms are represented as nodes, and the connections between them are represented as edges [16]. Central nodes and bridge nodes can be identified by network and centrality indexes. Central nodes are those most associated with other nodes and play the most important role in the network. Bridge nodes are those nodes with the strongest links between syndromes or communities; activation in one syndrome or community can be transferred to another syndrome or community [17, 18]. Thus, bridge symptoms can help to identify effective intervention targets for comorbidities [19].

Studies have examined the relationship between internet addiction and depression or anxiety using network analysis. For example, researchers conducted network analysis studies between internet addiction and depression, and found central symptoms such as preoccupation with the internet and neglect chores to spend more time online, and bridge symptoms such as sooth disturbing thoughts, anticipation, and suicide ideation [20–22]. Network analysis has also been conducted between internet addiction and anxiety [23] and revealed central symptoms such as academic decline due to internet use and depressed/moody/nervous only while being offline and bridge symptoms such as *feeling afraid* and *restlessness*. Only one study has examined the network among internet addiction, depression, and anxiety [24] and revealed central symptoms such as *restlessness* and *loss of control* and bridge symptoms such as *sleep disorders* and *tachycardia*. The study also revealed important edges such as “*prefer the excitement online*”—“*spend more time online over*,” and “*suicide ideation*”—“*guilty*” in the internet addiction and depression network [22].

Most of the previous studies focused on one age group; however, internet addiction, depression, and anxiety show variations in different age groups [25, 26]. From childhood to adolescence, the prevalence of internet addiction increased [27]. Children’s internet use may be more restricted under the guidance of parents and teachers [28], while adolescents are more independent and easily influenced by their peers [29]. Over‐reliance on the internet may lead to a disconnection from reality and then to negative consequences [30]. Therefore, depression and anxiety also became worse from children to adolescents [31]. In addition, the association between internet addiction and depression and anxiety may be different in children and adolescents. For example, in children, depressive symptoms are usually the “booster” of their internet addiction [32]. In adolescents, the association is mostly bidirectional. Facing multiple pressures such as academic and social, adolescents are prone to developing depression and anxiety and often turn to the internet to escape reality; in turn, long‐term internet use further exacerbates their emotional problems [33, 34]. However, the interaction between syndromes (internet addiction, depression, and anxiety) and the difference in network structure between children and adolescents remain unclear.

Taken together, the network structure among internet addiction, depression, and anxiety needs further examination; moreover, children and adolescents exhibited differential patterns of internet addiction, depression, and anxiety [25, 26], however, whether the interrelationship among these symptoms and network structure are different between children and adolescents remained unclear. To fill this gap in the literature, this study aimed to construct the network among internet addiction, anxiety, and depression in children and adolescents and compare the network structure between children and adolescents. We predicted there would be a stable network among internet addiction, depression, and anxiety in children and adolescents, and there would be similarities and differences between the networks in the two groups. By clarifying these issues, targeted intervention could be implemented in specific age groups.

## 2. Methods

### 2.1. Participants

This cross‐sectional survey was conducted in 2023 using an online questionnaire platform. The participants were primary and secondary school students recruited from schools in Beijing and Hebei. The questionnaires were administered collectively by class in school computer classrooms, thereby ensuring that participation was restricted to students from the participating schools. A total of 12,779 (6383 males and 6396 females) valid self‐report questionnaires were obtained. All participants were aged between 10 and 18 years. In the present study, participants aged 10–12 years (*n* = 6371) were categorized as children, whereas those aged 13–18 years (*n* = 6408) were categorized as adolescents for analytical purposes. This operational classification was based on developmental research and previous studies on Chinese samples. Specifically, ages 10–12 have been characterized as preadolescence [35], and previous research on Chinese youth has similarly classified individuals aged 9–12 years as children and those aged 13–18 years as adolescents [36]. This grouping is also broadly consistent with the evidence of substantial physical and neurodevelopmental changes across late childhood and adolescence [37, 38]. This study received approval from the Ethics Committee of Capital Normal University and was carried out in compliance with the Declaration of Helsinki. Informed consent was obtained from both students and their guardians.

### 2.2. Measures

The Internet Addiction Test (IAT) was used to measure internet addiction [39, 40]. This questionnaire includes 20 questions on internet use‐related scenarios. Participants were required to rate the frequency of these symptoms within a year (e.g., “*How often do you lose sleep due to late-night log-ins*?”) on a 5‐point scale (1 = “*rarely*,” 2 = “*occasionally*,” 3 = “*frequently*,” 4 = “*often*,” and 5 = “*always*”). The total score ranged from 20 to 100, with a higher total score indicating more severe internet addiction. In this study, Cronbach’s *α* was 0.938.

Anxiety was measured using the Chinese version of the seven‐item Generalized Anxiety Disorder‐7 (GAD‐7) scale [41, 42]. Participants were required to report how they felt over the past 2 weeks (e.g., “*Not being able to stop or control worrying*.”) on a 4‐point scale (0 = “*not at all*,” 1 = “*several days*,” 2 = “*more than half the days*,” and 3 = “*nearly every day*”). A higher total score indicates more severe anxiety symptoms. In this study, Cronbach’s *α* was 0.922.

Depressive symptoms were assessed with the Patient Health Questionnaire (PHQ‐9) [43, 44]. Participants were asked to report how they had felt in the past 2 weeks (e.g., “*Little interest or pleasure in doing things*”). They responded on a 4‐point scale (0 = “*not at all*,” 1 = “*several days*,” 2 = “*more than half the days*,” and 3 = “*nearly every day*”). A higher total score indicates more severe depression. In this study, Cronbach’s *α* was 0.906.

### 2.3. Statistical Analysis

#### 2.3.1. Sample Characteristics

Descriptive analysis was performed using SPSS 26.0, and independent sample *t*‐test and chi‐square test were used to compare demographics and questionnaire scores between the children and adolescents groups.

Network analysis included four parts: network estimation, centrality estimation, network accuracy and stability estimation, and network comparisons. The significance level was set to 0.05 for all analyses.

#### 2.3.2. Network Estimation

The network model was built and analyzed in R software (version 4.3.1) using the R package “qgraph” (version 1.9.2). We estimated a Gaussian graph model (GGM), and in order to reduce the number of pseudo edges and improve the interpretability of the results, we regularized the network model using the Least Absolute Shrinkage and Selection Operator (LASSO), a recognized regularization algorithm that eliminates weak associations by removing potential “false positive” edges from the model [16]. The extended Bayesian information criterion (EBIC) was used to select the best subset of neighbor factors for each node (symptom). In addition, the function “AverageLayout” in the R package “qgraph” was used to visualize different networks in the same layout [16]. In a network, symptoms are visualized as nodes, and interconnections between symptoms are represented as edges. The stronger the connection between symptoms, the closer they are to the network. The thickness of edges indicates the strength of the association between nodes, and the color of edges indicates the direction of the association (blue indicates positive correlation and red indicates negative correlation). The R package “mgm” was used to estimate the predictability of each node. Predictability is defined as the variance in the nodes explained by all other nodes in the network [45].

#### 2.3.3. Centrality Estimation

In network analysis, in order to quantify the centrality of nodes in the network, we calculated the centrality indexes using the function “centralityPlot” in the R package “qgraph” [46]. If there is no negative correlation in the network, it is more reliable to use strength as the core measure of centrality [47]. Given the presence of negative correlations in our network, this study used the EI as the centrality indicator.

Considering that our network consisted of three symptom communities—anxiety, depression, and internet addiction—bridging centrality was also calculated to quantify the extent to which symptoms of one community were linked to symptoms of the other communities [18]. The bridge function in the R‐package networktools (Version 1.2.3) was used for bridge centrality analysis [48]. In this study, we used the bridge expected influence (BEI) to indicate bridge centrality.

#### 2.3.4. Accuracy and Stability Estimation

Given that the estimated network model was subject to sampling variations, it was necessary to examine the accuracy and stability of the edge weights and centrality indexes [49]. The R package “bootnet” was used to evaluate the stability and accuracy of the network [16]. By resampling the data with replacements, the parameter estimates obtained from these resampled samples were used to build a 95% confidence interval (CI) to estimate the accuracy of the edges, with a narrower CI meaning higher accuracy [50].

The stability of the node properties was estimated by using the case deletion method. If most of the samples can be excluded from the data set and no significant changes in the node centrality indexes are observed, the network is considered stable. CIs of edge weights were estimated by the bootstrapping method. We estimated the central stability coefficient (CS coefficient) as the index, for which values greater than or equal to 0.25 indicated acceptable stability, while values greater than or equal to 0.5 indicated good stability [51].

#### 2.3.5. Network Comparisons

We used the Network Comparison Test (NCT) (version 2.2.1) to examine the differences in network structure between the two age groups (children vs. adolescents) with the permutation test (1000 permutations) [52]. The differences in network structure, the global strength, and each specific edge between groups were examined. False discovery rate (FDR) correction was used for multiple comparison adjustment [53].

## 3. Results

### 3.1. Sample Characteristics

A total of 12,779 individuals participated in the study, including 6371 (49.9%) children and 6408 (50.1%) adolescents. The demographic information and IAT, GAD‐7, and PHQ‐9 scores are presented in Table 1.

**Table 1:** Characteristics of participants.

Variable	Total (*N* = 12,779)	Children (*N* = 6371)	Adolescents (*N* = 6408)	*χ* ^2^/*t*	*p*
	*M* ± SD	*M* ± SD	*M* ± SD		
Male (*N*/%)	6383/49.9%	3106/48.8%	3277/51.1%	0.08^a^	0.777
Female (*N*/%)	6396/50.1%	3265/51.2%	3131/48.9%	—	—
Age	13.01 ± 2.22	11.14 ± 0.76	14.87 ± 1.54	173.49^b^	<0.001
IAT	39.71 ± 16.34	35.06 ± 14.97	44.33 ± 16.33	33.48^b^	<0.001
GAD‐7	4.50 ± 5.10	3.91 ± 5.00	5.09 ± 5.12	13.24^b^	<0.001
PHQ‐9	5.38 ± 6.08	4.53 ± 5.83	6.22 ± 6.21	15.92^b^	<0.001

*Note*: GAD‐7, generalized anxiety disorder scale; PHQ‐9, patient health questionnaire.

Abbreviations: IAT, internet addiction test; *M*, mean; SD, standard deviation.

^a^Indicates *χ* ^2^ value.

^b^Indicates *t* value.

### 3.2. Network Structure of IAT, GAD‐7, and PHQ‐9

Figure 1 shows the network structure of internet addiction, depression, and anxiety in all participants. In total, 335 edges were not zero (52%) among 648 possible edges. The predictability of symptoms is shown in the circular pie chart in Figure 1. The average predictability was 0.526, which indicated that, on average, 52.6% of the variance of each node could be explained by all other nodes in the model.

**Figure 1:**
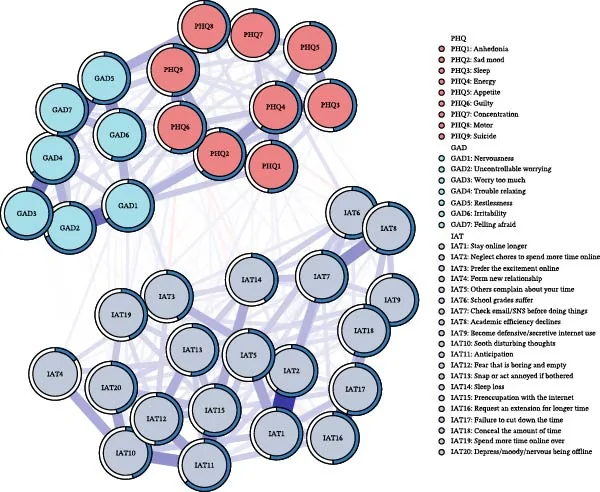
The network structure of anxiety, depression, and internet addiction symptoms in all participants (*N* = 12,779). Red nodes indicate symptoms of depression, blue nodes indicate symptoms of anxiety, and gray nodes indicate symptoms of internet addiction. A ring pie chart indicates predictability. More strongly connected symptom nodes in the graph are closer to each other, and thicker edges between symptoms indicate strong correlations.

The network model generally showed that nodes within the community were more strongly associated than nodes between communities; in addition, the connections between depression and anxiety was stronger than between internet addiction and depression and between internet addiction and anxiety, consistent with previous studies and suggesting that depression and anxiety belong to different clusters than internet addiction (i.e., internalizing problems and externalizing problems) [24].

### 3.3. Centrality Analysis

In terms of network centrality, node IAT11 (“*anticipation*”) had the highest EI, followed by node IAT2 (“*neglect chores to spend more time online*”) and node GAD2 (“*uncontrollable worrying*”) (Figure 2).

**Figure 2:**
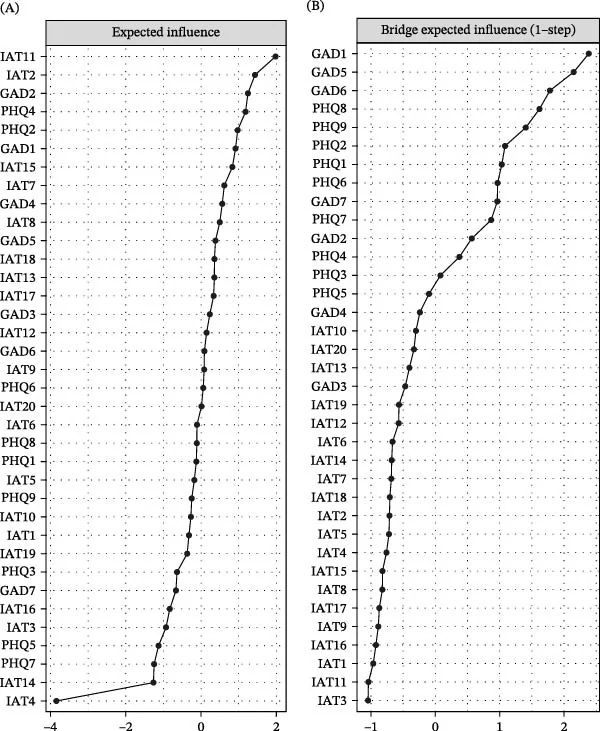
Centrality indexes of each item in all participants (*N* = 12,779): (A) expected influence (EI) and (B) bridge expected influence (BEI).

In terms of bridge centrality, node GAD1 (“*nervousness*”) had the highest BEI and was the most critical bridge symptom connecting the IAT, PHQ, and GAD communities. This was followed by node GAD5 (“*restlessness*”) and node GAD6 (“*irritability*”) (Figure 2).

### 3.4. Network Accuracy and Stability

The accuracy and stability of the network structure were good. Figure 3 presents the results of the stability of centrality indexes. The CS coefficient of the overall network was greater than 0.75, indicating that the network had excellent stability. The 95% CIs of edge weights were very narrow, indicating that the edges were accurate. The bootstrapping difference test showed that most of the comparisons of weights between edges were statistically significant. The results of the bootstrapped 95% CI for edge weights and bootstrapped differences test for edge weights are shown in Figures S1 and S2.

**Figure 3:**
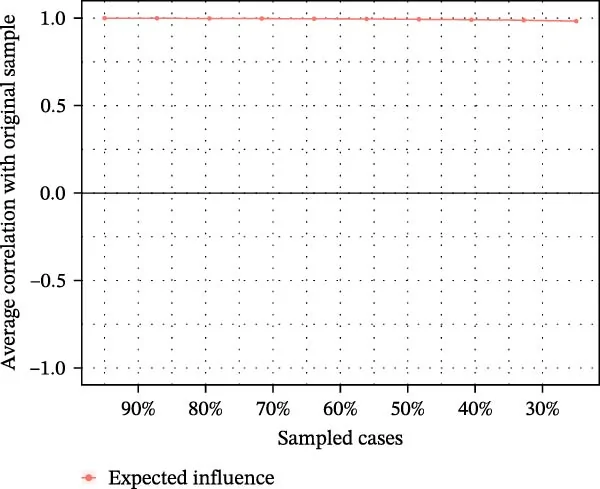
The stability of centrality indexes using case‐dropping bootstrapping in all participants (*N* = 12,779).

### 3.5. Network Comparisons

Regarding the comparison of networks between children and adolescents, we found no significant difference in the global strength of the network (*p* = 0.920), but a significant difference was found in network structure (*p* = 0.003). The network structure in different age groups was accurate and stable (Figures S3 and S4). We further compared the network structure between children and adolescents (Figure 4).

**Figure 4:**
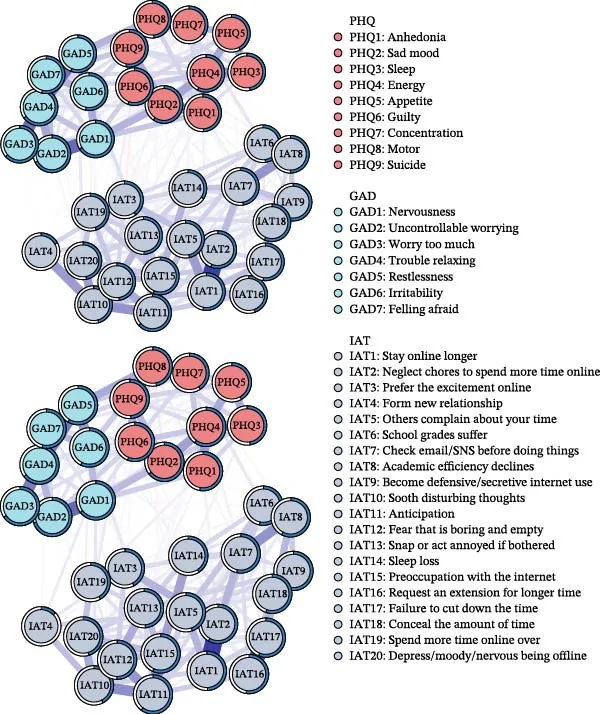
The network structure of internet addiction, depression, and anxiety in children (*n* = 6371) and adolescents (*n* = 6408).

#### 3.5.1. Centrality Comparisons of Networks Between Children and Adolescents

In terms of network centrality, node IAT11 (“*anticipation*”), node IAT2 (“*neglect chores to spend more time online*”), and node GAD2 (“*uncontrollable worrying*”) had the highest EI in both children and adolescents; therefore, they were the core symptoms for understanding the network model of internet addiction, depression, and anxiety among children and adolescents (Figure 5A).

Figure 5The comparison of centrality indexes of the network in children (*n* = 6371) and adolescents (*n* = 6408): (A) expected Influence (EI) in children and adolescents, (B) bridge expected influence (BEI) in children, and (C) bridge expected influence (BEI) in adolescents.

**Figure:**
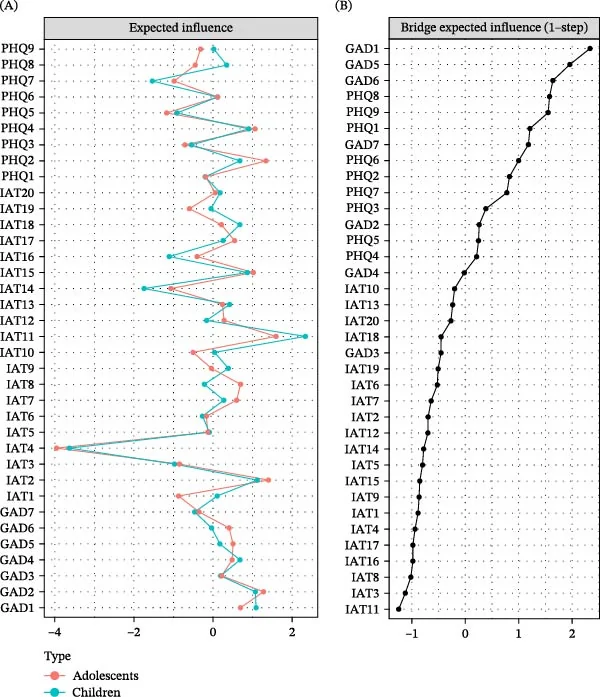

**Figure:**
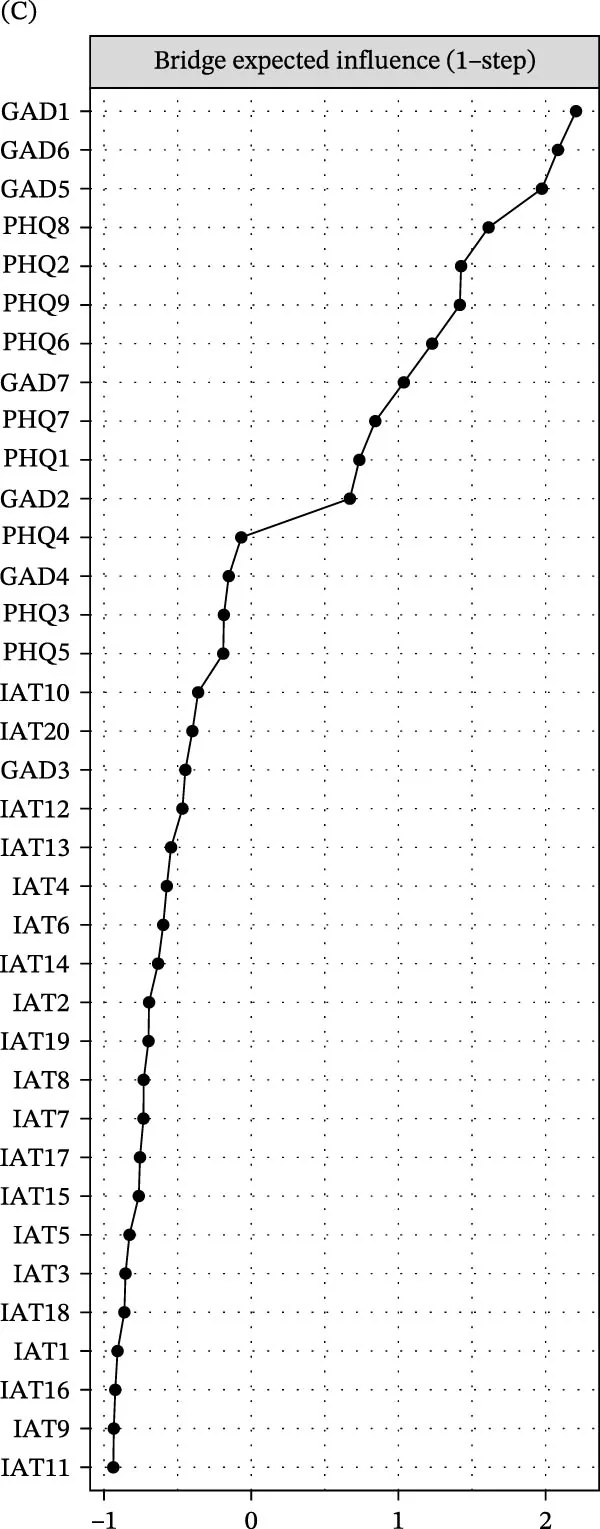

In terms of bridge centrality, the most important bridge nodes of the two networks were also the same. Node GAD1 (“*nervousness*”), node GAD6 (“*irritability*”), and node GAD5 (“*restlessness*”) had the highest BEI and were the key bridge symptoms connecting the IAT, PHQ, and GAD communities (Figure 5B,C).

#### 3.5.2. Differences in Network Structure Between Children and Adolescents

All edges with significant differences between children’s and adolescents’ networks are shown in Table S1. Here, we present the most significant within‐community and between‐community edges.

For group comparisons of edges within communities, the most significantly different edges in each community were as follows: IAT3‐IAT19 (“*prefer the excitement online*”—“*spend more time online over*”) (*p* < 0.001); GAD5‐GAD7 (“*restlessness*”—“*feeling afraid*”) (*p* < 0.01), and PHQ5‐PHQ9 (“*appetite*”—“*suicide*”) (*p* < 0.05). All these edges were stronger in adolescents than in children.

For cross‐community edges, the most significantly different edges between each two communities were as follows: PHQ5‐IAT10 (“*appetite*”—“*sooth disturbing thoughts*”) (*p*  < 0.001), GAD7‐IAT16 (“*feeling afraid*”—“*request an extension for longer time*”) (*p*  < 0.001), and PHQ8‐GAD2 (“*motor*”—“*uncontrollable worrying*”) (*p* < 0.01). All these edges were stronger in children than in adolescents.

## 4. Discussion

The present study examined the relationship between internet addiction, depression, and anxiety in children and adolescents using network analysis. The networks were stable and accurate. Children and adolescents showed similar central and bridge symptoms in the network; they also exhibited significant differences in the symptom network structure. Specifically, adolescents showed significantly stronger edges within communities than children, e.g., IAT3‐IAT19 (“*prefer the excitement online*”—“*spend more time online over*”), GAD5‐GAD7 (“*restlessness*”—“*feeling afraid*”), and PHQ5‐PHQ9 (“*appetite*”—“*suicide*”) (*p* < 0.05). Children exhibited stronger cross‐community edges of PHQ5‐IAT10 (“*appetite*”—“*sooth disturbing thoughts*”), GAD7‐IAT16 (“*feeling afraid*”—“*request an extension for longer time*”), and PHQ8‐GAD2 (“*motor*”—“*uncontrollable worrying*”) than adolescents.

### 4.1. Central Symptoms and Bridge Symptoms of the Network in Children and Adolescents

In both children and adolescents, the central symptoms with the highest EI were IAT11 (“*anticipation*”), IAT2 (“*neglect chores to spend more time online*”), and GAD2 (“*uncontrollable worrying*”). These results are consistent with previous studies that IAT2 was the central symptom of the internet addiction and depression network [20, 21], IAT11 was an important symptom in the internet addiction and depression network [21] and showed the highest outgoing expected influence in a cross‐lagged panel network analysis [54]. Studies suggested that anticipation/craving was one of the main characteristics of addiction, and this may cause emotional problems [55, 56]. Uncontrollable worry might lead to increased internet use as an escape, while excessive internet use can further exacerbate worry through social comparison, fear of missing out, etc.; in addition, worry and depression can intensify each other, leading to further withdrawal and isolation [57, 58]. These results highlight the core characteristics of the internet addiction, depression, and anxiety network, namely, the individual’s inability to extricate themselves from the internet and anticipation if not online [59]. This dependence not only affects the children and adolescents’ daily activities and learning [3] but may also lead to impaired social and emotional development [60].

Bridge symptoms include GAD1 (“*nervousness*”), GAD6 (“*irritability*”), and GAD5 (“*restlessness*”). These results are consistent with a previous study showing that the most significant bridge symptoms in the internet addiction‐anxiety network were symptoms from the anxiety community (e.g., GAD7 [*afraid*] and GAD5 [*restlessness*]), suggesting the role of anxiety in internet addiction. While the results are inconsistent with Shen et al. [24], in which the bridge symptoms were *sleep problems* and *tachycardia* in the network of internet addiction, depression, and anxiety, this might be because they used different measures from the present study and their study was conducted in left‐behind children. The abovementioned symptoms were the most influential bridge symptoms in both children and adolescents, suggesting the role of anxiety in the internet addiction, depression, and anxiety network in both groups. The compensatory internet use theory proposed that when individuals are faced with stress, anxiety, or other negative emotions, they may seek the internet as a means of compensation to alleviate these discomforts [54]. For children and adolescents, due to their relatively weak self‐regulation abilities, they may be more inclined to internet addiction when faced with negative emotions such as anxiety [58]. Therefore, we need to pay more attention to bridge symptoms and intervene in time to reduce their adverse effects on comorbidity.

### 4.2. Differences Between Children and Adolescents in the Network

Although the central symptoms and bridge symptoms were similar across the two groups (children and adolescents), an in‐depth network comparison revealed significant differences in the network structure (e.g., edge weights) between children and adolescents. These edges play a crucial role in connecting different symptoms within or between communities/comorbidity of disorders. By analyzing these edges, we can gain a clearer insight into the differences in the network among children and adolescents and thus provide a strong basis for prevention and intervention efforts.

Specifically, we observed a stronger association between IAT3 (“*prefer the excitement online to the time with others*”) and IAT19 (“*spend more time online over going out with others*”) in adolescents than in children. This result reveals a specific pattern of adolescents’ internet use behavior. They are more inclined to seek stimulation, satisfy curiosity, and engage in social interaction through the internet rather than through real‐life social interaction; this may be due to multiple factors such as academic pressure, identity exploration, and social needs [39]. Adolescents are in a critical period of physical and mental development, and they face more complex challenges and pressures than children [61]. The internet provides a convenient and diversified platform to meet their needs, particularly in social communication and entertainment [1]. However, over‐reliance on the internet may also lead them to ignore real‐life social interactions and experiences, further exacerbating the risk of internet addiction [25].

Adolescents exhibited a stronger GAD5 (“*Restlessness*”)—GAD7 (“*feeling afraid*”) association than children. Adolescents face multiple challenges such as physical development, academic pressure, and interpersonal relationships compared with children [62, 63], each of these factors can lead to increased anxiety about the future. When a person feels afraid, his/her body goes into “fight or flight” mode. This triggers the release of hormones like adrenaline and cortisol, which can lead to physical symptoms like restlessness. At the same time, when our body is restless, it can feel uncomfortable and unsettling; this can trigger feelings of anxiety and fear [64]. In addition, the adolescent brain is still developing, particularly in the areas responsible for decision‐making and emotional control, which makes adolescents more susceptible to external environments and more prone to experiencing heightened emotions and a lack of control, leading to a stronger link between restlessness and fear than in children [65].

We also found that the connection between PHQ5 (“*appetite*”) and PHQ9 (“*Suicide ideation*”) was significantly stronger in adolescents than in children. In adolescents, a stronger connection between PHQ5 and PHQ9 may mean that they are more prone to suicidal ideation when faced with appetite problems. Adolescent suicidal ideation or self‐harm was significantly associated with a loss of appetite [66]. There are many reasons that affect appetite, such as hormonal fluctuations, increased learning pressure, and the complexity of social environments [67]. At the same time, the focus on body image during adolescence is also an important factor affecting their appetite and mood [68, 69]. At this stage, they become more concerned with their appearance and status among peers, which can lead to excessive preoccupation with weight and diet [70]. Portrayals of ideal bodies in the media and social networks also put them under great pressure [71]. When adolescents are unhappy with their bodies, they may develop emotional eating conditions such as a loss of appetite or overeating, which further affects their mental health [72]. When negative emotions and stress accumulate to a certain extent, they may lead to suicidal ideation [73].

For cross‐community edges, PHQ5 (“*appetite*”)—IAT10 (“*sooth disturbing thoughts*”) showed stronger connection in children than in adolescents. It might be that in childhood, when emotion regulation and coping strategies are not well‐developed, children may rely more on external stimuli to regulate emotions, such as food or the internet, than adolescents [74]. At the same time, as an important entertainment and social communication tool in children’s lives nowadays, the internet becomes an interesting place to attract their attention when children have troubles and helps them temporarily divert their attention [12], thus indirectly alleviating behaviors such as loss of appetite or overeating. Individuals may turn to the internet to escape stress and distress in real life, but such escape behavior often only provides temporary relief, which may lead to more serious mental health problems in the long run [75].

IAT16 (“*request an extension for longer time*”)—GAD7 (“*feeling afraid*”) exhibited a stronger association in children than in adolescents. Children are often sensitive to the opinions and attitudes of their parents, and they may fear being criticized or punished for behaving in ways that do not meet their parents’ expectations or standards [76]. This fear may lead them to develop feelings of anxiety or afraid when online [77] and try to avoid potential negative consequences by limiting their online time. For example, they may tell themselves; “just play for a little while” when online as a self‐regulation strategy. This lack of control may lead to more anxiety and strengthen the bidirectional relationship between internet addiction and anxiety [58]. In contrast, adolescents may show greater independence and autonomy [78] and may be less sensitive to parental criticism or punishment than children. They may have more clear judgments and decisions about their online behavior [79]. This may weaken the association between these two nodes in adolescents compared with children.

The connection of PHQ8 (“*motor*”)—GAD2 (“*uncontrollable worrying*”) was stronger in children than in adolescents. Children’s brains, particularly the frontal lobe, which is responsible for higher cognitive functions and emotional regulation, are less mature than those of adolescents [80]. Therefore, they may have difficulty understanding and regulating emotions as effectively as adolescents in the face of stressors such as studying and getting along with teachers and peers [81]. Children may express their anxiety through physical symptoms like slowed movement or excessive fidgeting, as they could not articulate their worries effectively [82]. They might also struggle to control these physical reactions, leading to stronger links between these behaviors and worrying. Therefore, in the process of intervention and treatment, we must fully consider the differences in age and symptom characteristics and provide programs for different age groups to solve mental health problems more effectively.

### 4.3. Limitations and Future Directions

There are some limitations in this study. First, the cross‐sectional design allowed us to characterize group‐level associations among symptoms but did not permit the examination of within‐person symptom dynamics or individual‐level heterogeneity over time. Individuals with similar levels of symptom severity may exhibit different temporal patterns of symptom interactions [83]. Future studies using longitudinal or intensive repeated‐measures designs are needed to examine the temporal dynamics of symptoms and to determine whether these dynamics differ between children and adolescents. Second, the sample was limited to Beijing and Hebei, and future studies should recruit participants from a wider geographical area and different socioeconomic contexts [84] to improve the universality and generalization of the results. Third, this study relied on self‐reported data, which may be subject to subjective bias [85], future studies can recruit participants with diagnosis. Fourth, several socioeconomic, family, and academic factors, such as family income, parental marital status, parental occupation, parent–child relationships, and academic performance, were not assessed in the present study. Therefore, their potential influence on the observed relationships could not be accounted for. Future studies should incorporate these factors to further examine the robustness of the present findings. Finally, the operational age cutoff used to distinguish children (ages 10–12) from adolescents (ages 13–18) was somewhat arbitrary, and this may have influenced the observed network differences between the two groups. Future studies should examine whether these symptom network structures remain consistent across alternative developmental age cutoffs or when age is modeled as a continuous variable.

### 4.4. Implications

Notwithstanding the above limitations, this study has implications. The findings revealed similarities and differences in the network structure across different age groups, providing a scientific basis for the development of targeted prevention and intervention strategies [86]. In addition, through data analysis of a large sample, the study ensured the robustness of the results [87], providing a reliable basis to support policymaking.

## 5. Conclusion

To conclude, we found that *anticipation* (IAT11), *neglect chores to spend more time online* (IAT2), and *uncontrollable worrying* (GAD2) were central symptoms in the symptom networks of internet addiction, depression, and anxiety in both children and adolescents, while *nervousness* (GAD1), *irritability* (GAD6), and *restlessness* (GAD5) served as bridge symptoms in both groups. Adolescents exhibited stronger within‐community edges than children, whereas children exhibited stronger between‐community edges than adolescents. Understanding the similarities and differences in these network structures may help inform the development of targeted interventions that better address the needs of children and adolescents.

## Funding

This research was supported by the Key Project of Beijing Education Science Planning, Reevaluation and Cognitive Training to Treat College Students with Internet Addiction (Grant BEAA21046) and Beijing Digital Education Research (Grant BDEC2023619098).

## Conflicts of Interest

The authors declare no conflicts of interest.

## Supporting Information

Additional supporting information can be found online in the Supporting Information section.

## Supporting information


**Supporting Information** The Supporting Information provides additional results on network comparisons, edge‐weight differences, estimation accuracy, and network stability. Table S1: All edges showing significant differences between the children’s and adolescents’ networks. Figure S1: The bootstrapped 95% confidence intervals for edge weights, while Figure S2: The results of bootstrapped difference tests between edge weights. Figure S3: The stability of network estimates across age groups. Figure S4: The accuracy of edge‐weight estimates within each age group. Together, this Supporting Information provides additional information for evaluating the reliability and robustness of the network findings.

## Data Availability

The data that support the findings of this study are available upon request from the corresponding author. The data are not publicly available due to privacy or ethical restrictions.
