# Supplementary material for: The Relationship Among Internet Addiction, Depression, and Anxiety in Children and Adolescents: A Network Analysis
Source: Depress Anxiety. 2026 Nov 1;2026:3015380. doi: 10.1155/da/3015380 (PMC13725119; doi:10.1155/da/3015380)
Supplement: Supplementary file 1 [file DA-2026-3015380-s001.docx]

**Supplementary Materials Description**

This supplementary materials document contains additional tables and figures that support the findings presented in the main manuscript. The contents include:

Table S1:

Displays all edges with significant differences between children's and adolescents’ networks.

Figure S1:

Presents the bootstrapped 95% confidence intervals (CI) for the edge weights in the network analysis. The results demonstrate the precision of the estimated edge weights across the network.

Figure S2:

Shows the results of the bootstrapped differences test for edge weights. It highlights the significant differences between edge weights, providing insights into the variations in network connections.

Figure S3:

Illustrates the stability of the network structure across different age groups. The results confirm that the network structure remains reliable, supporting the robustness of our findings.

Figure S4:

Demonstrates the accuracy of the network structure in different age groups. The results validate the precision of the network model, ensuring that the observed patterns are statistically sound.

**Table S1.** All significantly different edges between children and adolescents

| Var1 | Var2 | p-value | Var1 | Var2 | p-value | Var1 | Var2 | p-value |
| --- | --- | --- | --- | --- | --- | --- | --- | --- |
| PHQ4 | IAT6 | 0.000999 | IAT14 | IAT17 | 0.00999 | IAT6 | IAT8 | 0.032967 |
| PHQ5 | IAT10 | 0.000999 | PHQ7 | IAT3 | 0.010989 | IAT20 | GAD1 | 0.032967 |
| IAT3 | IAT19 | 0.000999 | IAT3 | IAT6 | 0.011988 | IAT15 | IAT17 | 0.033966 |
| IAT7 | IAT20 | 0.000999 | IAT11 | GAD4 | 0.012987 | PHQ6 | IAT11 | 0.034965 |
| IAT16 | GAD7 | 0.000999 | PHQ5 | IAT7 | 0.013986 | IAT3 | GAD2 | 0.034965 |
| PHQ8 | IAT2 | 0.001998 | IAT2 | IAT9 | 0.013986 | PHQ5 | GAD7 | 0.034965 |
| PHQ8 | IAT7 | 0.002997 | PHQ5 | IAT1 | 0.015984 | IAT18 | GAD7 | 0.034965 |
| IAT16 | IAT17 | 0.002997 | PHQ6 | GAD2 | 0.015984 | IAT17 | IAT20 | 0.036963 |
| PHQ1 | IAT18 | 0.002997 | PHQ5 | PHQ9 | 0.016983 | IAT3 | GAD3 | 0.037962 |
| PHQ8 | GAD2 | 0.002997 | IAT11 | IAT18 | 0.016983 | PHQ8 | IAT1 | 0.03996 |
| IAT20 | GAD2 | 0.002997 | PHQ3 | GAD7 | 0.017982 | IAT6 | IAT7 | 0.040959 |
| IAT1 | IAT9 | 0.003996 | PHQ3 | IAT9 | 0.018981 | PHQ2 | GAD2 | 0.040959 |
| IAT4 | IAT10 | 0.003996 | IAT6 | IAT15 | 0.01998 | GAD1 | GAD4 | 0.040959 |
| IAT8 | IAT17 | 0.003996 | PHQ6 | PHQ9 | 0.025974 | IAT11 | IAT12 | 0.044955 |
| PHQ4 | GAD5 | 0.003996 | IAT20 | GAD5 | 0.025974 | IAT5 | IAT17 | 0.044955 |
| IAT1 | GAD6 | 0.003996 | IAT10 | GAD1 | 0.027972 | IAT14 | GAD2 | 0.045954 |
| GAD5 | GAD7 | 0.004995 | PHQ7 | IAT5 | 0.028971 | PHQ9 | IAT16 | 0.046953 |
| IAT17 | IAT19 | 0.005994 | PHQ9 | IAT4 | 0.02997 | IAT3 | IAT20 | 0.046953 |
| IAT12 | IAT19 | 0.006993 | IAT2 | IAT18 | 0.02997 | PHQ7 | IAT9 | 0.048951 |
| IAT2 | IAT20 | 0.006993 | PHQ9 | IAT20 | 0.02997 | IAT11 | IAT20 | 0.048951 |
| IAT2 | IAT15 | 0.00999 | PHQ3 | IAT20 | 0.030969 | GAD4 | GAD7 | 0.048951 |

Fig. S1 The accuracy of the bootstrapped 95% CI for edge weights in all participants (N=12779).

Fig. S2 Bootstrap difference test for edge weights in all participants.

**Children**

**Adolescents**

Fig. S3 The stability of centrality indexes using case-dropping bootstrapping in children (upper panel) and adolescents (lower panel).

Fig. S4 The accuracy of the bootstrapped 95% CI for edge weights in children (left panel) and adolescents (right panel).

**Adolescents**

**Children**
